# Do Unresectable Giant Cell Tumors of Bone Treated With Denosumab Progress After Discontinuation of Treatment?

**DOI:** 10.1002/cnr2.70117

**Published:** 2025-01-11

**Authors:** DENO Research Group, Carolina de la Calva, Manuel Angulo, Paula González‐Rojo, Ana Peiró, Pau Machado, Juan Luis Cebrián, Roberto García‐Maroto, Antonio Valcárcel, Pablo Puertas, Gregorio Valero‐Cifuentes, Óscar Pablos, Miriam Maireles, María Luisa Fontalva, Iván Chaves, Aida Orce, Luis Coll‐Mesa, Israel Pérez, Fausto González, María del Carmen Sanz, Isidro Gracia

**Affiliations:** ^1^ Hospital Universitario y Politécnico La Fe Valencia Spain; ^2^ Hospital de la Santa Creu i Sant Pau Barcelona Spain; ^3^ Hospital Clínico San Carlos Madrid Spain; ^4^ Hospital Clínico Universitario Virgen de la Arrixaca Murcia Spain; ^5^ Hospital Universitario de Bellvitge L'Hospitalet de Llobregat Spain; ^6^ Hospital Universitario Nuestra Señora de Candelaria Santa Cruz de Tenerife Spain; ^7^ Hospital Universitario Ramón y Cajal Madrid Spain

**Keywords:** denosumab, giant cell tumors of bone, recurrence, unresectable

## Abstract

**Background:**

Denosumab represents a valuable treatment option for unresectable giant cell tumors of the bone (GCTBs). However, no standardized protocols exist determining the length of administration, with few studies having been published on patients who reached the end of treatment.

**Aims:**

To analyze the outcomes of patients diagnosed with GCTB and who had finished single treatment with denosumab.

**Methods and Results:**

This is a multicenter, retrospective, descriptive study carried out in seven Spanish hospitals with multidisciplinary sarcoma and musculoskeletal tumor boards, between 2009 and 2019. Sixteen patients diagnosed with unresectable GCTBs and treated with denosumab who had reached the end of their treatment were recruited for the study and had been followed up for a minimum of 2 years. Fifty percent of patients discontinued denosumab after showing signs of tumor control. The disease remained stable in 69% of patients (*n* = 11), with a median recurrence‐free survival time of 46 months (20–157 months) after being treated for a median period of 19 months (5–83 months). Four patients experienced local progression, and one presented multifocal progression. These five patients were treated for a median period of 46 months (14–76 months), with a median recurrence‐free survival time of 9 months (5–25 months).

**Conclusion:**

The findings of the present study suggest that discontinuation of denosumab in patients with unresectable GCTB is not necessarily associated with the progression of the disease. Further research is needed to determine how long denosumab should be administered to minimize the risk of recurrence.

## 1 Introduction

Giant cell tumors of the bone (GCTBs) are a category of locally aggressive, rarely metastasizing neoplasms [1, 2, 3, 4, 5]. The disease is typically treated surgically through intralesional curettage and wide excision. However, curettage techniques are associated with high recurrence rates (15%–50%) and wide excision tends to constitute a very aggressive alternative, with considerable morbidity and loss of function [1, 2, 3, 4, 5, 6, 7].

Denosumab is a human monoclonal antibody with high affinity to RANKL (receptor activator of nuclear factor kappa B ligand), which prevents interaction of RANKL with the RANK receptor of osteoclast precursors, thus decreasing bone resorption. The drug was FDA‐approved and EMA‐approved in 2013 and 2014, respectively, for unresectable GCTBs or when surgical resection would likely result in high morbidity, and thus, it has mainly been used as neoadjuvant treatment in aggressive types of GCTB or as a single therapy in nonoperable tumors [1, 2, 3, 4, 5, 6, 8, 9, 10].

Although several authors have reported promising results for denosumab as a single treatment, with some of them even suggesting a curative effect, there is currently insufficient evidence in the literature regarding the required length of treatment or whether it must be administered for life. Moreover, there is a dearth of studies analyzing the evolution of patients after discontinuation of treatment due to either the implementation of a rest period or the emergence of associated adverse events [1, 11, 12, 13].

The purpose of this study was to analyze the outcomes of patients diagnosed with GCTB and who had finished single treatment with denosumab.

## 2 Methods

This is a multicenter, retrospective, descriptive analysis of the use of denosumab in the context of nonoperable GCTBs. The study was approved by the La Fe University Hospital's Ethics Committee and the Spanish Agency of Medicines and Medical Devices (AEMPS) (approval code JFT‐DEN‐2019‐01). The study was performed by the DENO research group, which is part of the Spanish Musculoskeletal Tumors Research Consortium (LIETAL). All patients were duly informed about the risks and objectives of the study and provided written informed consent.

We selected patients who had a pathological diagnosis of unresectable—due to being inoperable or because the possible surgery was highly aggressive and would result in significant morbidity for the patient—GCTB at one of seven Spanish hospitals with multidisciplinary tumor boards, between 2009 and 2019. Patients were required to have received and discontinued single treatment with denosumab following the standard administration regimen (subcutaneous administration of 120 mg a month, with extra doses at Days 8 and 15 during the first month supplemented with calcium and vitamin D [2.500 mg and > 400 IU, respectively] to prevent hypocalcemia), with a minimum follow‐up period of 2 years after drug discontinuation. Patients under the age of 18, as well as pregnant women, persons with systemic phosphocalcic metabolism disorders preceding their GCTB diagnosis, and patients previously treated with denosumab, were excluded from the study.

A record was made of the patients' anthropometric data, as well as of the reason why the treatment was indicated and of the origin, location, and severity of the tumor, according to Campanacci et al.'s classification [14]. During the first 2 years of treatment with denosumab, patients underwent radiological monitoring every 3 months with local CT and MRI, as well as chest CT, with a semi‐annual frequency for the following 3 years and annually thereafter. Both CT and MRI scans were assessed to evaluate patients' radiological response according to the Inverse Choi Density/Size (ICDS) criteria, categorizing results as full or partial response, stable disease, progression of disease, or impossible to evaluate [15]. Clinical response was considered favorable if patients reported progressive pain relief in the tumor area over a period of at least 14 days, and unfavorable if they reported a progressive increase of pain in the tumor area for a period of at least 14 days. Both the radiological and clinical responses collected in the article were based on the best responses patients showed during their follow‐up period [12].

Patients who did not exhibit tumor progression following discontinuation of denosumab were analyzed to determine their progression‐free survival while off treatment. The group where the tumor did progress after withdrawal of the drug was examined to determine the length of time patients remained free of recurrence after discontinuation. An analysis was made in both groups of the length of time during which patients had received the drug.

The reasons for discontinuation as well as any adverse events and their degree of toxicity were also examined, using the Common Terminology Criteria for Adverse Events (CTCAE—Version 5.0) [16].

All statistical analyses were conducted using Stata Statistical Software release 16 (*StataCorp. 2019. College Station, TX: StataCorp LLC*.). A descriptive statistical analysis was performed of all the data, which was presented as mean (SD), median (range or interquartile range [IQR]) or percentages. The Kaplan–Meier method was used to estimate recurrence‐free survival following discontinuation of denosumab.

## 3 Results

**FIGURE 1:**
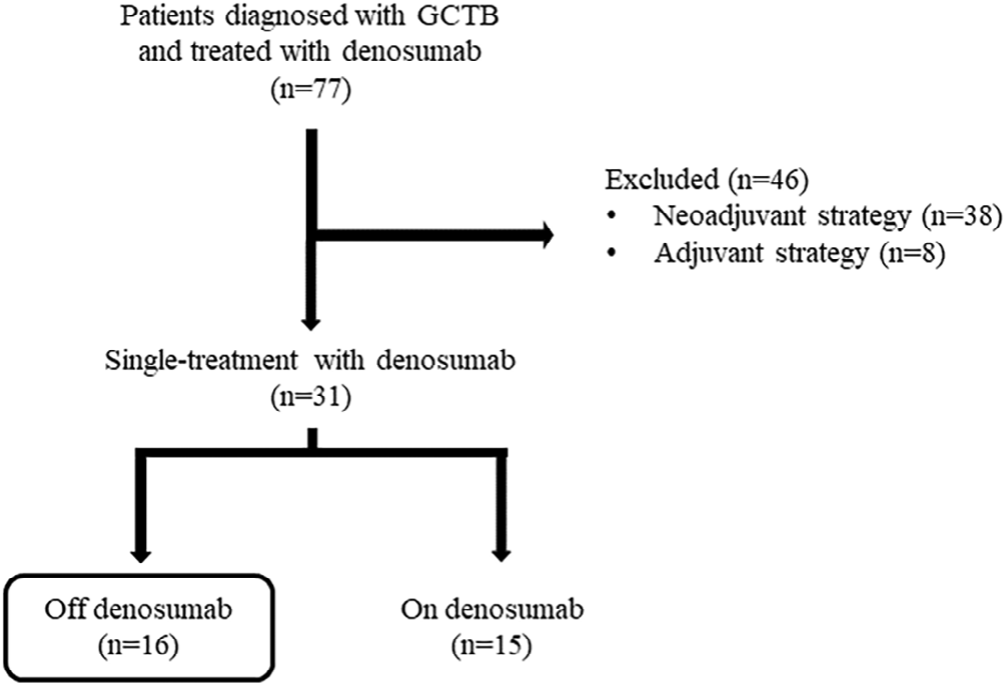
Patient selection process.

Sixteen patients were recruited for the study, all of whom presented with localized, metastasis‐free GCTBs (Figure 1). Their anthropometric characteristics are summarized in Table 1. The patients' clinical and radiological response during the administration of denosumab is shown in Table 2. A comprehensive description of the individual patient characteristics can be found in Appendix I.

**TABLE 1:** Clinical and anthropometric characteristics of the subjects in the study.

Characteristic	*N* = 16
Sex	
Men	5 (31%)
Women	11 (69%)
Age (years)	39.7 ± 14.0 [18–65]
Origin	
Primary	8 (50%)
Recurrence	8 (50%)
Tumor location	
Axial skeleton	3 (19%)
Distal radius	4 (25%)
Pelvis	2 (13%)
Distal femur	2 (13%)
Proximal tibia	4 (25%)
Talus	1 (6%)
Campanacci's radiological classification	
I	1 (6%)
II	9 (56%)
II with fracture	1 (6%)
III	5 (31%)

**TABLE 2:** Clinical and radiological response to administration of denosumab.

	*N* = 16
Clinical response	
Favorable	16 (100%)
Unfavorable	0
Radiological response—ICDS criteria	
Full response	2 (13%)
Partial response	6 (38%)
Stable disease	6 (38%)
Progression of disease	0
Impossible to evaluate	2 (13%)

Patients were followed up for a median time of 4 years (2–13 years) after the completion of their treatment. A total of 11 patients (68.8%) had not recurred after completion of treatment with denosumab. Their scans did not show any sign of lytic lesions and remained stable. The median length of administration of the drug was 19 months (5–83 months), and the median length of progression‐ or recurrence‐free survival time after discontinuation of the drug was 46 months (20–157 months).

Four patients experienced local recurrence, and one presented multifocal recurrence in the axial skeleton. All of them received denosumab for longer than a year, with a median length of administration of 46 months (14–76 months). They all experienced a recurrence within the first 2 years after discontinuation, with a median recurrence‐free survival time after ending treatment of 9 months (5–25 months). The patients who suffered multifocal recurrence relapsed only 6 months after discontinuation of denosumab, which they had been treated with for 26 months.

Three of these patients had discontinued treatment due to toxicity. Following their recurrence, they underwent different treatment approaches: one received a single surgery, another received a single treatment with denosumab, and the third underwent a combined surgical and radiotherapy strategy, with denosumab introduced subsequently. Although at the beginning of their treatment course, these patients did not undergo surgery as it was considered too aggressive an option, following their recurrence and due to compelling reasons, it had to be employed. The remaining two patients had discontinued denosumab due to a rest period after achieving favorable outcomes. Upon recurrence, they received a single treatment with denosumab, one of whom also underwent percutaneous ablation. Currently, all five patients remain stable with no signs of tumor progression.

**FIGURE 2:**
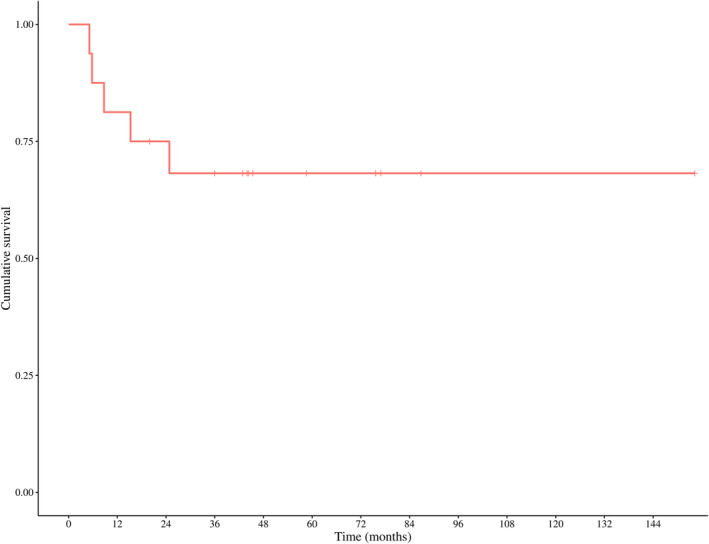
Kaplan–Meier recurrence‐free survival curve.

The Kaplan–Meier estimated recurrence‐free survival rate following cessation of denosumab was 81.2% (95% CI, 64.2–100.0) at 12 months, 75.0% (95% CI, 56.5–99.5) at 24 months, and 68.2% (95% CI, 48.6–95.7) at 36 months (Figure 2).

The reasons for discontinuation of denosumab are presented in Table 3. They include adverse events (38%), implementation of a rest period due to effective tumor control (50%), and a personal decision by the patient (13%). The median length of administration of denosumab in patients where a rest period was implemented was 40 months (9–83 months).

**TABLE 3:** Reasons for discontinuation of denosumab.

	*N* = 16
Toxicity	6 (38%)
Fatigue	2
Mandibular osteonecrosis	2
Muscle weakness	1
Limb pain	2
Hypertransaminasemia	1
Asthenia	1
Insomnia	1
Neutropenia	1
Rest period	8 (50%)
Clinical‐radiological stability	6
Full response	2
Patient's personal decision	2 (13%)

Although denosumab was well‐tolerated by most patients, nine of them presented with adverse events including fatigue (3), mandibular osteonecrosis (3), muscle weakness (2), diarrhea (1), limb pain (4), back pain (1), hypertransaminasemia (1), asthenia (1), myalgia (1), anemia (1), insomnia (1), and neutropenia (1). There were no cases of hypophosphatemia, nausea, dyspnea, hypocalcemia, headache, leg cramps, cough, or eczema, nor were there any Grade > 3 adverse events recorded. Only two patients reported Grade 3 adverse events, one of them presenting with mandibular osteonecrosis and insomnia and the other with neutropenia. In both cases, a decision was made to discontinue the treatment.

No cases of death occurred during the course of the study.

## 4 Discussion

The use of denosumab in the context of GCTB is typically reserved for patients with lesions where surgery is not viable or would result in a significant functional loss for the patient. However, the dearth of clinical trials and multicenter studies with enough patients to draw meaningful conclusions means that no specific treatment protocols have been developed. This study reports on a group of patients with GCTB who completed treatment with denosumab.

Some authors have suggested that treatment with denosumab may only be effective while the drug is administered due to the cytostatic effect it produces in tumor cells, and that its discontinuation could inevitably lead to recurrence [6, 17, 18]. The results of our analysis appear to contradict this suggestion, as the vast majority of our patients remained recurrence‐free for a median period of 46 months after completing treatment, with only four experiencing a local recurrence and one experiencing multifocal recurrence, which is a rare event. A total of 69% of our patients did not show progression of the disease, a rate within the range reported by Bukata et al., whose patients had an 85% progression‐free survival with a median follow‐up time of 69.1 months [12], and the 74% and 60% reported by Chawla et al. [19]. and Palmerini et al., whose patients had a median follow‐up time of 65.8 and 15 months [13], respectively. The progression‐free rate after discontinuation was above 60% in all cases. To the best of our knowledge, the mechanisms underlying progression in only certain cases after drug discontinuation remain unknown.

Median recurrence‐free survival following completion of treatment with denosumab is highly variable, with authors reporting lengths of survival from 8 [13] to 23 [12] and even 39 [19] months. Our own recurrence‐free length of survival also fell within that range, with a median of 9 months. Chawla et al. [19] estimated a recurrence rate after discontinuation of denosumab of 23.6% at 1 year, 42.3% at 2 years, and 45.9% at 3 years. Our analysis also found the same increasing risk of recurrence from the first to the third year, where our estimated recurrence risk was estimated at 31.8%. The data indicate that progression can occur beyond the 2‐year established as a limit in the literature. There is currently some controversy over the time at which treatment with denosumab should be discontinued, the most usual reasons for discontinuation being the development of toxicity or the implementation of a rest period due to improvement or long‐term tumor stabilization [20]. Comparisons with the work of other authors in this regard are difficult as the literature on the subject is basically made up of incidental reports. Chawla et al.'s clinical trial [19], Bukata et al.'s subanalysis [12], and Palmerini et al.'s 10‐patient series [13] treated subjects for longer than in this analysis, with mean treatment periods ranging from 3.6 to 4.5 years. However, the mean length of treatment in Thomas et al. [21] and Ueda et al. [22] was approximately 6 months, and Rutkowski et al. [23] treated their patients for a mean of 8 months. The disparity in the literature comes openly to light when analyzing the mean length of treatment implemented in all the different articles published to date, which ranges between 4 and 54 months [12, 13, 17, 19, 20, 21, 22, 23, 24, 25, 26, 27, 28], also in line with the variability found in our analysis, where mean values stood between 5 and 83 months.

On the other hand, the literature seems to suggest an association between longer administration times and a higher risk of local progression [29]. This was borne out by the results of our study, as patients who did not recur had received the treatment for a median of 19 months and those who did had been treated for 46 months. Given the disparity in the sample sizes of both groups (11 vs. 5 patients), no hard‐and‐fast conclusions could be drawn as to whether any statistically significant differences existed with respect to the length of administration of denosumab.

Currently, there are no guidelines on how to schedule treatment in cases where patients respond well over a prolonged period. In the past few months, some authors have proposed implementing de‐escalations by increasing treatment intervals to around 12 weeks, which seems to have worked in short patient series [30], yielding similar tumoral control as the monthly treatment [31]. Moreover, it has been suggested that patients could benefit from periods of “drug holidays” while being closely monitored using bone turnover biomarkers, such as Urinary N‐telopeptide, serum C‐telopeptide or Tartrate‐resistant acid phosphatase 5b [32].

Ideally, significant differences should be found between patients with progression of the disease and those where the disease remained stable after discontinuation with respect to clinical data, reason for stopping denosumab, or length of treatment. However, the group of patients in our study with progression of the disease after discontinuation is made up of just five patients, which is too little to provide any meaningful statistical power.

Although half of our patients developed some kind of treatment‐related adverse event, the safety profile of denosumab was in general terms acceptable. The benefits of the treatment and the potential risk of developing an adverse event were examined individually for each case and, eventually, the decision was made to discontinue denosumab in six of the eight patients that developed an adverse event. No life‐threatening adverse events were identified, and the toxicity rates obtained appeared to be lower than those reported elsewhere. This was probably due to the shorter treatment period in our study, several authors having found higher toxicity levels in patients receiving long‐term regimens of denosumab [12, 13, 19].

The main limitations of this study are related to the small number of patients recruited, the retrospective nature of data collection, and the fact that the group of patients exhibiting tumor progression after discontinuation was made up of only five individuals. These circumstances make it impossible to support a recommendation regarding the appropriate length of treatment with denosumab, making further research into the subject of crucial importance. However, precisely due to this low recurrence rate, we are able to provide new evidence supporting the benefits of denosumab in the treatment of GCTB, even after its discontinuation. Additionally, we believe we have presented an accurate reflection of the clinical guidelines followed across Spain.

In a nutshell, discontinuation of denosumab in patients with unresectable GCTBs is not necessarily related to the progression of the disease. Further studies are required to determine the length of time during which patients must be treated to allow the implementation of a rest period associated with the lowest possible recurrence risk.

## Author Contributions

Conceptualization: C.C., M.A., P.G.R., and I.G. Methodology: DENO Research Group. Software: DENO Research Group. Validation: M.A., C.C., and I.G. Formal analysis: C.C. and P.G.R. Investigation: C.C. and P.G.R. Data curation: C.C. and P.G.R. Writing – original draft preparation: C.C. and P.G.R. Writing – review and editing: DENO Research Group. Supervision: C.C., M.A., and I.G. All authors have read and agreed to the published version of the manuscript.

## Ethics Statement

The study was approved by the La Fe University Hospital's Ethics Committee and the Spanish Agency of Medicines and Medical Devices (AEMPS) (approval code JFT‐DEN‐2019‐01).

## Conflicts of Interest

The authors declare no conflicts of interest.

## Data Availability

The data that support the findings of this study are available on request from the corresponding author. The data are not publicly available due to privacy or ethical restrictions.

## Appendix I Detailed Characteristics of the 16 Patients Included in the Study

### I.1

**Table:**

Sex	Age (years)	Tumor location	Origin	Campanacci's radiological classification	Denosumab administration (months)	Clinical response	Radiological response	Reason for discontinuation	Time without treatment (months)	Recurrence	Time until recurrence (months)
Female	49	Pelvis	Primary	II	46	Favorable	Partial response	Toxicity	37	No	—
Female	56	Distal radius	Recurrence	II	62	Favorable	Stable disease	Rest period	44	No	—
Female	34	Talus	Recurrence	II	6	Favorable	Stable disease	Toxicity	88	No	—
Female	53	Pelvis	Recurrence	II	5	Favorable	Stable disease	Personal decision	45	No	—
Female	36	Distal femur	Recurrence	III	12	Favorable	Full response	Rest period	157	No	—
Female	48	Proximal tibia	Recurrence	II	17	Favorable	Full response	Rest period	78	No	—
Male	23	Axial skeleton	Primary	II	43	Favorable	Partial response	Personal decision	60	No	—
Male	22	Pelvis	Primary	II	19	Favorable	Partial response	Rest period	45	No	—
Male	34	Distal femur	Primary	II	9	Favorable	Stable disease	Rest period	46	No	—
Female	50	Distal radius	Recurrence	III	83	Favorable	Impossible to evaluate	Rest period	20	No	—
Male	48	Axial skeleton	Primary	III	43	Favorable	Partial response	Toxicity	77	No	—
Male	28	Axial skeleton	Primary	III	25	Favorable	Impossible to evaluate	Toxicity	30	Yes	6
Female	26	Proximal tibia	Recurrence	II	61	Favorable	Stable disease	Rest period	50	Yes	16
Female	58	Distal radius	Recurrence	II with fracture	14	Favorable	Partial response	Toxicity	79	Yes	5
Female	34	Axial skeleton	Primary	III	76	Favorable	Partial response	Rest period	37	Yes	9
Female	18	Pelvis	Primary	I	46	Favorable	Stable disease	Toxicity	25	Yes	25
